# Mechanical, Corrosion and Biological Properties of Room-Temperature Sputtered Aluminum Nitride Films with Dissimilar Nanostructure

**DOI:** 10.3390/nano7110394

**Published:** 2017-11-17

**Authors:** Cristina Besleaga, Viorel Dumitru, Liliana Marinela Trinca, Adrian-Claudiu Popa, Constantin-Catalin Negrila, Łukasz Kołodziejczyk, Catalin-Romeo Luculescu, Gabriela-Cristina Ionescu, Razvan-George Ripeanu, Alina Vladescu, George E. Stan

**Affiliations:** 1 National Institute of Materials Physics, RO-077125 Magurele, Romania; cristina.besleaga@infim.ro (C.B.); viorel.dumitru@infim.ro (V.D.); liliana.trinca@infim.ro (L.M.T.); adrian.claudiu@gmail.com (A.-C.P.); catalin.negrila@infim.ro (C.-C.N.); 2 Army Centre for Medical Research, RO-010195 Bucharest, Romania; 3 Institute of Materials Science and Engineering, Lodz University of Technology, 90-924 Lodz, Poland; lukasz.kolodziejczyk@p.lodz.pl; 4 National Institute for Lasers, Plasma and Radiation Physics, RO-077125 Magurele, Romania; catalin.luculescu@inflpr.ro; 5 Petroleum-Gas University of Ploiesti, RO-100680 Ploiesti, Romania; ionescug@upg-ploiesti.ro (G.-C.I.), rrapeanu@upg-ploiesti.ro (R.-G.R.); 6 National Institute for Optoelectronics, RO-077125 Magurele, Romania; alinava@inoe.ro

**Keywords:** aluminum nitride, magnetron sputtering, structure, nano-mechanical tests, corrosion, biocompatibility

## Abstract

Aluminum Nitride (AlN) has been long time being regarded as highly interesting material for developing sensing applications (including biosensors and implantable sensors). AlN, due to its appealing electronic properties, is envisaged lately to serve as a multi-functional biosensing platform. Although generally exploited for its intrinsic piezoelectricity, its surface morphology and mechanical performance (elastic modulus, hardness, wear, scratch and tensile resistance to delamination, adherence to the substrate), corrosion resistance and cytocompatibility are also essential features for high performance sustainable biosensor devices. However, information about AlN suitability for such applications is rather scarce or at best scattered and incomplete. Here, we aim to deliver a comprehensive evaluation of the morpho-structural, compositional, mechanical, electrochemical and biological properties of reactive radio-frequency magnetron sputtered AlN nanostructured thin films with various degrees of *c*-axis texturing, deposited at a low temperature (~50 °C) on Si (100) substrates. The inter-conditionality elicited between the base pressure level attained in the reactor chamber and crystalline quality of AlN films is highlighted. The potential suitability of nanostructured AlN (in form of thin films) for the realization of various type of sensors (with emphasis on bio-sensors) is thoroughly probed, thus unveiling its advantages and limitations, as well as suggesting paths to safely exploit the remarkable prospects of this type of materials.

## 1. Introduction

In the last years, Aluminum Nitride (AlN) became a hot topic material for many sensing applications. This is due to its good piezoelectric properties, large bandgap (6.2 eV), high temperature stability (up to 1000 °C), chemical inertness, compatibility with the process technology, that makes AlN a very interesting material for realization of various types of sensors [1,2,3,4,5,6,7]. For instance, piezoelectric AlN thin films were successfully integrated in pressure sensors capable to operate in high temperature or in harsh environments conditions (e.g., combustion pressure sensors for cars) [8] or for measuring pressure fluctuations in rotating machinery such as compressors and turbines [9]. Furthermore, AlN-based vibration sensors for the monitoring of industrial machines [10] or for ultra-high temperatures applications [11], ultra-violet (UV) sensors suitable for operating in high temperature/harsh environment conditions [12], as well as various pyroelectric sensors [13,14,15], have been also investigated. Thin film piezoelectric AlN (on Si) gravimetric resonant [16] as well as surface acoustic wave (SAW) [17] based gas sensors, were also demonstrated.

Compatibility with complementary metal–oxide–semiconductor (CMOS) processing made AlN very attractive for realization of micro-electro-mechanical system (MEMS) sensors like accelerometers [18,19], gyroscopes [20], piezoelectric microphones for aircraft fuselage noise sources identification [21], or for implantable hearing aid applications [22]. AlN-based pressure, temperature and 3-axis acceleration sensors integrated on a single chip were recently attempted [23].

The advent of the era of biosensors generated a special focus on the feasibility of electronics applied in biomedical and biotechnology sectors. Attempts are constantly made to apply and link the progresses of nowadays nano- and micro-electronics with the sensitivity, selectivity and complexity of biological systems and processes. Ineluctably, AlN, due to its remarkable electronic properties, is one of the materials envisaged to serve as multi-functional sensing platform. The fabrication of flexible piezoelectric pressure sensors using AlN thin films deposited on polymeric substrates have been reported [24], together with their use for measuring human muscle movements [25] and monitoring the respiration and heartbeat during sleep [26]. AlN thin films have been recently employed to realize piezoelectric micro-machined ultrasonic transducers used for gesture recognition [27], intravascular ultrasound imaging [28], or ultrasonic fingerprint sensing [29]. Also, AlN is seen a very promising material for realization of various microfluidic and biosensing applications, with integrated lab-on-chip diagnostic systems being envisaged [30]. For instance, AlN thin-film bulk acoustic resonator (FBAR) devices have been used to detect carcinoembryonic antigen (cEA) [31], or human immunoglobulin E (IgE) antibody [32]. Furthermore, a AlN/diamond based FBAR for biomedical applications was endeavored [33], whilst other researchers suggest the great promise of AlN thin films for biosensing of cell differentiation [34].

The current deposition method of choice for synthesizing piezoelectric AlN thin films for electronic and opto-electronic applications is reactive magnetron sputtering (MS), operating either in direct-current [35,36], pulsed direct-current [37,38], or radio-frequency [39,40,41] regimes. The advantages derive from the ability of MS techniques to produce high quality dense, adherent and large-area uniform films [42]. The features of sputtered layers can be engineered by tailoring the deposition conditions [42]. The survey of scientific literature unveils that the synthesis of highly *c*-axis textured AlN films can be achieved by MS for the most part in low total sputtering pressure (i.e., 0.2–0.5 Pa [43,44,45,46,47,48]) and nitrogen-in-argon dilution conditions (i.e., 20–50%) [43,44,45,46,47,48] (with the exception of pulsed direct-current regime where higher nitrogen volumetric concentrations are needed (i.e., >50%) [47]); whereas, the target-to-substrate separation distance, substrate temperature and target powder densities vary significantly in the ranges 20–120 mm, 80–650 °C, and 0.018–0.150 W/mm^2^ [43,44,45,46,47,48], respectively. A new emerging solution for growing high quality *c*-axis oriented AlN is the two-step sputtering which implies the insertion at the substrate—prospect AlN coating interface of a thin AlN seed layer synthesized at an elevated temperature (e.g., 450 °C), followed by the deposition at a lower temperature of the bulk AlN layer [41,49]. Nevertheless, it is imperative for the (i) compatibility and integrability with the lift-off process (to the difference of chemical etching, in the case of lif-off the selectivity is not an issue) and/or (ii) development of flexible electronic devices, to achieve good quality AlN films at low temperatures (i.e., 30–80 °C), due to technological restrictions such as the low softening points of polymeric substrates, typically situated below 100 °C. However, one should take into account that in the case of a reduced deposition temperature (compatible with both the polymeric substrate and CMOS fabrication technologies) the synthesis of high quality films becomes challenging. If highly *c*-axis textured AlN films are required for realization of piezoelectric sensors and devices (the piezoelectric properties being strongly correlated with the crystalline quality and *c*-axis texturing), the AlN films with lower crystallinity could be employed for other topical applications (e.g., gate-dielectrics layers for thin films transistors, metal-insulator-metal capacitors or other electronic devices on large area flexible substrates), where crystallinity and preferred crystallite orientation is not as important as the delineation of a low-temperature and low-cost deposition process, compatible with the large-area heat-sensitive substrates.

Besides the AlN films intrinsic piezoelectricity, essential for obtaining high performance sustainable sensors are also the surface morphology and mechanical performance (elastic modulus, hardness, wear, scratch and tensile resistance to delamination, adherence to the substrate). Also, in order to fully exploit the great potential of AlN films for biomedical applications, cytocompatibility and corrosion resistance have to be explored. To date, there are only scarce and incomplete assessments of the mechanical [50], corrosion [51] and cytocompatibility [34,52,53] of AlN sputtered thin films. 

Thereby, the main objective of this work was to conduct a comprehensive evaluation of the physical-chemical and functional (with emphasis on the nano-mechanical, corrosion resistance and cytocompatibility properties) performance of reactive radio-frequency magnetron sputtered (RF-MS) AlN films synthesized at room-temperature, having dissimilar structural quality starting from different base pressure levels, in view of probing their potential suitability for the realization of various type of electronic devices (implantable sensors, included).

## 2. Materials and Methods 

### 2.1. Thin Film Synthesis

For the deposition of AlN thin films, a high-purity (5N) Al target (Mateck GmbH, Jülich, Germany) having a diameter of 110 mm and thickness of 3 mm was used. The Si^++^ (100) substrates, with resistivity in the range of 5 × 10^−4^ to 10^−3^ Ω·cm (Ossila, Sheffield, UK), were successively cleaned ultrasonically in acetone and iso-propanol for 10 min and then dried in an argon flow.

The films synthesis experiments were carried out with an UVN-75R1-type deposition system (Vacma, Soviet Union), equipped with planar magnetron guns (cathodes), a RF generator of 1.78 MHz and a rotary pump-oil diffusion pump vacuum system. A condensation cold trap has been used to avoid the backstreaming of oil molecules and with that to reduce the contamination of the reaction chamber (vacuum vessel).

Prior to deposition, the substrates were etched for 10 min in argon (Ar) plasma (0.3 Pa) produced by a wolfram plasmatron by applying a 0.4 kV DC bias voltage and a power of ~200 W, in order to remove the potential remnant impurities. Earlier studies have evidenced the positive role of this procedure on the adherence of the sputtered films [54,55,56]. The process was optimized in the past; longer etching times or higher plasma power value leading to morphological irregularities on substrate surface, which are unfavorable to an optimal growth of a film with minimized internal tensions. The complete elimination of the native SiO*_x_* layer cannot be accomplished in the given etching conditions, but only in ultra-high vacuum by repeated sessions of argon ion sputtering and flash annealing [57]. However, due to the preferential sputtering occurring in the given etching conditions, which favor the removal of the lighter species, at least an oxygen depleted substrate surface will be attained. Such a surface will be readily active toward incoming sputtered species (in this case Al and N) which can lead to a stronger chemical bonding of the coating to the substrate, as shown in previous works [54,55,56].

Furthermore, preceding films synthesis, the cathode target was pre-sputtered in a two-stage process: (i) cleaned for 15 min in pure Ar (0.3 Pa), followed by a (ii) longer sputtering (60 min) in the actual working conditions (Table 1) in which the AlN films will be deposited. Such a target pre-sputtering process is essential for obtaining reproducible AlN films, as it enables first a clean metallic Al target surface (without native oxides) and then a stabilized sputtering deposition process.

The deposition was then carried out at a target power density of ~0.008 W/mm^2^ and a total gas pressure of 0.2 Pa, in a reactive atmosphere of Ar and N_2_ (with a volumetric concentration of N_2_ of 30%). The substrates were not intentionally heated during deposition. Their temperature, was only dependent on plasma self-heating, and reached a maximum of ~50 °C in the case of the longer depositions (as monitored with a build-in controller). The total gas flow was kept constant at 40 sccm. The deposition rates were estimated by spectroscopic ellipsometry measurements at ~14 nm/min and did not vary significantly with the initial base pressure attained in the reactor chamber. Based on the estimated deposition rate, AlN films with thicknesses of ~1, 1.5, or 3 of μm were deposited.

The sputtering pressure and working atmosphere conditions have been chosen based on our previous experience, when found optimal for the growth of *c*-axis textured films. They are, in many respects, similar to kindred works in this research field [43,44,45,46,47,48]. A lower sputtering pressure was preferred as it assures an increased kinetic energy transfer from plasma to the growing films surface [48], thus accommodating good crystalline quality. To attain low substrate temperature (in the absence of cooling facilities for the substrate holder), primarily a low power density should be employed, with complementary fairly large target-to-substrate distance. Although, there are reports of AlN films deposited by MS at “low-temperature” or “room-temperature,” in fact this should be rather interpreted as “deposition room-temperature,” with substrate indeed not intentionally heated, but still reaching 100–300 °C when using the typical moderate-to-high electrical powers [46,47,58]. In our study, we have decided to carry out the films deposition at a low power density (i.e., ~0.008 W/mm^2^) to prevent substrate overheating, but at a relatively short target-substrate separation distance (i.e., 30 mm) such as to attain a high deposition rate (i.e., ~14 nm/min). It is known that in the case or reactive sputtering a high deposition rate is reducing the incorporation of impurities from the background gases into the growing film [48]. Furthermore, a high deposition rate is also appealing, as it enables shorter fabrication times and thus economic efficiency. However, for well-textured *c*-axis AlN films the deposition rate should be maintained under 35 nm/min, such as to prevent the occurrence of stoichiometry and structural faults [48].

For the reactive magnetron sputtering deposition method, the base pressure at which the deposition chamber is initially evacuated (known as the “contamination level”) should play a decisive role in the synthesis of films with properties suitable for electronics and optoelectronics applications. Here, a systematic study on the influence of the base pressure (Table 1) on the *c*-axis texturing degree of sputtered AlN films and their derived overall features, was realized.

In view of their characterization and testing, sample batches of 25 specimens were prepared for each type of deposition condition to accommodate different investigations and assays. Prior to analysis the samples were stored in low humidity conditions at room-temperature (RT), in desiccator.

### 2.2. Morphological, Structural, Compositional, Mechanical, Optical and Electrical Characterization Methods

(i) Spectroscopic ellipsometry was employed to determine thicknesses of the AlN films. The measurements were performed using a Woollam Variable Angle Spectroscopic Ellipsometer (Lincoln, NE, USA) in the 1.2–4 eV spectral range, step 0.01 eV, at 3 angles of incidence: 45°, 60° and 75°.
(ii) The surface morphology of the deposited films was examined by atomic force microscopy (AFM), in non-contact mode using an NT-MDT NTEGRA Probe NanoLaboratory system (Moscow, Russia). A high-resolution silicon NT-MDT NSG01 cantilever (Moscow, Russia) was used, having a tetrahedral tip, with the last 500 nm from tip apex cylindrical. The typical curvature radius is 6 nm (at most 10 nm guaranteed by the producer). The tip height is 14–16 nm, the cone angle at the apex is 7–10°, whilst the aspect ratio is up to 7:1. The topographical AFM images were scanned over areas of 2 × 2 μm^2^ and the root mean square roughness (R_RMS_) has been inferred.
(iii) The crystallographic structure of each film was analyzed by X-ray diffraction (XRD) on a Bruker-AXS D8 Advance diffractometer (Karlsruhe, Germany) in parallel beam setting, with Cu K_α_ (λ = 1.5418 Å) radiation. The diffraction data were collected in symmetric (θ–θ) geometry in the angular range 2θ = 20°–80°, with 0.04° step size and 5 s acquisition time per step. The angular dispersion of the *c*-axis of the AlN crystallites with respect to the normal to the substrate has been evaluated by rocking curves (RC) measurements.
(iv) The analysis of the bonding configuration and molecular vibrations of AlN films was performed by Fourier Transform Infrared (FTIR) spectroscopy, using a Perkin Elmer Spectrum BX apparatus (Waltham, MA, USA) in transmission mode. The spectra were collected over a range of 4000–400 cm^−1^ by recording 128 individual scans at a resolution of 4 cm^−1^. Complementary, micro-Raman analyses have been conducted with a JASCO NRS-7200 Raman microscope (Oklahoma City, OK, USA) and 532 nm incoming laser radiation. The scattered photons were dispersed by an 1800 lines/mm grating monochromator and simultaneously visualized by a CCD camera.
(v) The compositional analyses have been performed by X-ray photoelectron spectroscopy (XPS), using a Specs GmbH (Berlin, Germany) equipment. XPS constitutes a powerful tool whose sensitivity enables to stress variations in the chemical environment of atoms (Al, N, O and C in this case), offering qualitative and quantitative information on the surface composition of the films and configuration of chemical bonds and chemical states. The photo-emission studies have been carried out at a pressure of ~10^−8^ Pa, using monochromatic Mg K_α_ (1253.6 eV) radiation, whilst the X-ray source was set at 300 W. A Phoibos electrons analyzer with a radius of 150 mm, operated at a pass energy of 50 eV for survey spectra and 5 eV for high-resolution scans of core electron levels of interest, has been employed. The sample neutralization during the measurements was achieved by using a flood gun, with an acceleration energy of 1 eV and an emission current of 1 mA. The resolution in terms of full width at half maximum (FWHM) is 0.45 eV. High-resolution scans were performed for the Al 2p, N 1s, O 1s and C 1s core levels. In order to remove inherent surface contaminants owned to environmental/adventitious carbonaceous species and other impurities, an in-situ Ar^+^ ions etching (using a Specs IQE11/35 ion gun) session has been performed for 5 min, at an energy of 3 keV and a pressure of 1 × 10^−3^ Pa. The ion currents were in the range of 5 µA due to chamber geometry, Ar pressure and low power of the gun. In the case of S4 optimized sample, deep-profiling analyses were performed for the quantification of the contamination level in the film’s volume. The following analysis sequence has been employed:
  - Non-etched sample measurements;
  - Etching stage I—5 min, 3 keV Ar^+^, 1 × 10^−3^ Pa, followed by measurements;
  - Etching stage II—60 min, 3 keV Ar^+^, 1 × 10^−3^ Pa, followed by measurements;
  - Etching stage III—60 min, 3 keV Ar^+^, 1 × 10^−3^ Pa, followed by measurements;
  - Etching stage IV—80 min, 3 keV Ar^+^, 1 × 10^−3^ Pa, followed by measurements.

One should note that a 3 keV and higher acceleration voltage for the Ar^+^ ions is usual in XPS depth profiles experiments for AlN and nitride materials, due to their higher surface bonding energy [59,60,61,62]. The etching conditions were established after a long-time experience gained in depth profile characterization studies in the field of nitride or hard films [63,64,65,66]. We evaluate the risk of unwanted chemical reactions as very low in such experimental conditions.

The XPS spectra were fitted with Voigt lines after a typical Shirley baseline correction procedure.

(vi) The adherence to the substrate of AlN films was estimated by the “pull-out” method using a standardized measurement instrument PAT_handy_ (maximum pull force = 1 kN) (DFD Instruments, Kristiansand, Norway) equipped with stainless steel testing elements having a diameter of Φ = 2.8 mm. The testing elements were glued on the films’ surface with a cyanoacrylate monocomponent adhesive (Epoxy E900S, DFD Instruments, Kristiansand, Norway). Then, the samples were placed for glue curing in a stabilized oven (Venticell, München, Germany) for 1 h, at a temperature of 130 °C. The detachment of the testing elements was achieved by the gradual increase of the pull-out force by means of a hydraulic head until fracture occurred [67,68]. The adherence values were inferred by a statistical calculation based on the ratio between the value of the recorded force and the area of the detached film surface, in accordance with the ASTM D4541 and ISO 4624 standards. The tests were done in quintuplicate. The adherence values are given as mean ± standard deviation.
(vii) The nano-mechanical properties of the AlN films have been explored by specific nano-indentation, nano-scratch and nano-wear tests, using an Agilent Nano Indenter (model G200) apparatus (Santa Clara, CA, USA). The machine was situated in a temperature (21–22 °C) and humidity (35–45%) controlled laboratory. The nano-indentation tests were performed in continuous stiffness measurement (CSM) mode with a diamond Berkovich tip (Micro Star Technologies, Huntsville, TX, USA). The system calibration was performed using a fused silica reference. The data were analyzed using the Oliver and Pharr [69] approach. The nano-scratch and nano-wear were investigated using a nanoindenter equipped with diamond conical tip of radius of 1 µm and an apex angle of 90°. The wear tests were carried out at a force value of 6 mN for 400 cycles, sliding distance of 100 μm and frequency of 1 Hz. All tests were performed under ambient conditions. The thermal drift threshold requirement for performing the nano-mechanical (hardness and modulus) and wear assays was set at 0.05 nm/s and 5 nm/s, respectively (in agreement with the manufacturer recommendations).

### 2.3. Corrosion Tests

The corrosion performance of the AlN layers was evaluated according to ISO 16429:2004(E) standard: “Implants for surgery—Measurements of open-circuit potential to assess corrosion behavior of metallic implantable materials and medical devices over extended time periods.” Two testing mediums were used: (i) an isotonic aqueous 0.9% (mass fraction) sodium chloride (NaCl) solution (thus a purely inorganic solution), as recommended by the ISO standard and in (ii) Dulbecco’s Modified Eagle’s Medium with 10% fetal bovine serum (DMEM-FBS) (Sigma Aldrich, St. Louis, MO, USA), which better simulates the complex organic-inorganic composition of the true intercellular fluid [70].

A VersaSTAT3 (Princeton Applied Research—Ametek, Princeton, NJ, USA) potentiostat and an ASTM G5 electrochemical cell were employed for monitoring the corrosion behavior of AlN/Si^++^ layers. The electrochemical cell used for the corrosion measurements consisted of a glass vessel, a capillary Haber-Luggin tube, a clamp-holder for capillary tube and three electrodes: a Pt counter electrode, a saturated calomel (SCE) as a reference electrode and the sample as a working electrode. A Teflon sample holder with 1 cm^2^ hole was employed to provide a controlled sample surface in direct contact with the testing liquid. The capillary tube was located in front of sample hole. A circulating bath thermal regulator (FA90) was used to maintain the temperature at a homeostatic value (i.e., 37 °C). To prevent contamination all vials were sterilized by dry-heating (180 °C/h). 

During the tests, each solution was stirred continuously using a magnetic stirrer (300 rpm) in order to eliminate the formation of hydrogen bubbles. 

The open-circuit potential (OCP) was monitored for 1 h, immediately after immersion in the electrolyte. Then, the linear polarization was recorded from −10 mV to +10 mV at a scan rate of 0.1667 mV/s, in order to determine the polarization resistance (R_p_). The Tafel curves were acquired by applying a ±250 mV polarization around the corrosion potential, with the scan rate of 0.1667 mV/s, in order to determine the corrosion potential (E_corr_), corrosion current densities (i_corr_) and corrosion rates (C_rr_). At the end, the polarization curves were recorded by sweeping the potential from −1 V to 1 V at a scan rate of 0.1667 mV/s. i_corr_ was calculated by graphical extrapolation of the anodic and cathodic branches of Tafel curves. R_p_ values were calculated from the Tafel curves as the slope of the potential versus current density plot at i = 0. C_rr_, in mm/year, was calculated using the following equation:

(1) $$\mathrm{Crr}=\frac{i_{\mathrm{corr}}K\cdot E_{W}}{A\cdot \rho }$$

where i_corr_ (given in µA/cm^2^), K is a constant that defines the units for the corrosion rate (0.003272 for corrosion rate given in mm/year), E_W_ is the equivalent weight in grams/equivalent, ρ is the density in g/cm^3^ and A is the sample area in cm^2^.

### 2.4. Biocompatibility Assessments

The biocompatibility assays have been performed complying with ISO 10993-5:2009 (“Biological evaluation of medical devices—Part 5: Tests for in vitro cytotoxicity”), using a procedure refined in Reference [68,71].

#### 2.4.1. Cell Cultures

For the AlN thin films biocompatibility tests, the Hs27 cell-line (ATCC, Manassas, VA, USA) was used. Cells were grown in a 5% CO_2_ humidified atmosphere incubator, at 37 °C, using the recommended cell culturing medium (DMEM with L-glutamine), with the following supplements: streptomycin (100 µg/mL final concentration), penicillin (100 UI/mL final concentration) and 10% bovine fetal serum.

Sample preparations: uncoated and S2 and S4 coated Si square samples with an area of 100 mm^2^ were cleaned with ethanol (70% and absolute) and dry-heat sterilized (180 °C/h). The sterile samples were transferred in laminar flow hood for cell cultures in sterile 24-wells cell plates. 

Cell seeding: after reaching confluence, the cells were detached with trypsin, transferred into sterile polyethylene 15 mL centrifuge tubes and centrifuged 10 min at 250× *g* after trypsin inhibition. 

The supernatant was discarded and fresh complete growing medium was added. The re-suspended cells were counted using a Bürker-Türk counting chamber, in order to adjust the cell “concentration” to 10^5^ cells/mL. A volume of 100 µL DMEM containing 10^4^ cells was placed on each sample. The plates were transferred for 5 h in the humidified atmosphere incubator. After adhering of cells, a volume 400 µL of fresh growing medium was added in each well. The cells have grown in the incubator for 24 h. After 24 h the cells were used for morphology, proliferation and cell death investigation, as presented hereunder.

All cell culture reagents used were produced by Sigma Aldrich (St. Louis, MO, USA).

#### 2.4.2. Cell Morphology

Cells were seeded onto samples (i.e., Si substrates, S2 and S4 AlN films) and examined by fluorescence imaging, in order to observe if morphology modifications appear upon growing on the surface of AlN structures. After fixation with para-formaldehyde (4% in phosphate-buffered saline—PBS, for 15 min at RT) the cells were washed thrice with PBS. Actin cytoskeleton was marked with phalloidin-AlexaFluor546 (Invitrogen, Carlsbad, CA, USA), cells being incubated for 1 h at RT, with a 100 µL of fluorochrome solution prepared accordingly to the producer specifications. The samples were washed thrice for 15 min, with PBS to remove the fluorochrome excess. The cells nuclei were counterstained with 4′,6-diamidino-2-phenylindole (DAPI) (Sigma Aldrich, St. Louis, MO, USA). After DAPI incubation (15 min at RT with a 1 µg/mL solution), cells were washed two times with PBS and a last time with double-distilled water. Samples were mounted with fluorescence mounting medium (Invitrogen, Carlsbad, CA, USA) and 0.17 mm thick glass coverslips. Cell morphology was examined with a fluorescence microscope with appropriate filters (Zeiss Axioplan, Jena, Germany). 

#### 2.4.3. Proliferation Assay

The capacity of cells to grow and proliferate onto the surface of S2 and S4 samples was investigated by 3-(4,5-dimethylthiazol-2-yl)-5-(3-carboxymethoxyphenyl)-2-(4-sulfophenyl)-2H-tetrazolium (MTS) assay (Promega, Madison, WI, USA). The procedure we used is presented in our previous publications [68,71]. In brief, after 24 h of growing, the cell culture medium was discarded and a volume of 400 µL of fresh phenol red free DMEM was introduced in each well. After 30 min necessary for cell accommodation, 80 µL of ready-to-use MTS reagent was added in each well. After 1 h of reaction in the incubator, the plates were gently shake on an orbital shaker (150 rpm/min). From each well 120 µL of medium was transferred into 96 microplates. Optical densities were read using a multimode microplate reader, (Zenyth 3100—Anthos, Beckman Coulter, Brea, CA, USA), at 490 nm. For results quantification, the experiment used a scale that was produced additionally with different number of Hs27 cells and a control of fresh complete cell growing media.

#### 2.4.4. Cell Toxicity Assay

Cell death from sample toxicity was investigated by lactate dehydrogenase (LDH) activity assay (kit produced by Thermo Scientific, Waltham, MA, USA). Solutions were prepared according to manufacturer instructions. Twenty-four hours after seeding, a volume of 50 µL of the supernatant medium, from each sample, was transferred into 96-wells plates. LDH substrate solution was added (50 µL in each well) and the plates were transferred into the incubator for 30 min. Further, 50 µL of stop buffer was added in each well. The optical densities were read using a multimode micro-plate reader, (Zenyth 3100—Anthos, Beckman Coulter, Brea, CA, USA). The results for each situation is given by subtracting the optical density value at 620 nm from the optical density values at 490 nm. For results quantification, the experiment used a scale that was produced additionally, with different number of Hs27 cells and a control of fresh complete cell growing media.

#### 2.4.5. Statistical Analysis

For statistics, all experiments were carried out in triplicate. To assess statistical significance, the unpaired Student’s *t*-test was used and a *p* < 0.05 was considered as significant.

## 3. Results and Discussion

### 3.1. Morphological Analyses

The AFM analyses (2 μm × 2 μm regions) revealed smooth AlN films surfaces having a reduced R_RMS_ roughness, situated in the range ~1.3–3.4 nm (see Table 2). We note that the similar R_RMS_ values have been inferred from larger scanning areas, i.e., 5 μm × 5 μm and 10 μm × 10 μm (data not shown), which testifies for the uniform smoothness of the films surface. Such low surface R_RMS_ are characteristic to AlN films obtained by reactive magnetron sputtering [44,45,46]. The films consisted of a uniform and compact matrix composed of tightly-packed grains.

**Figure 1:**
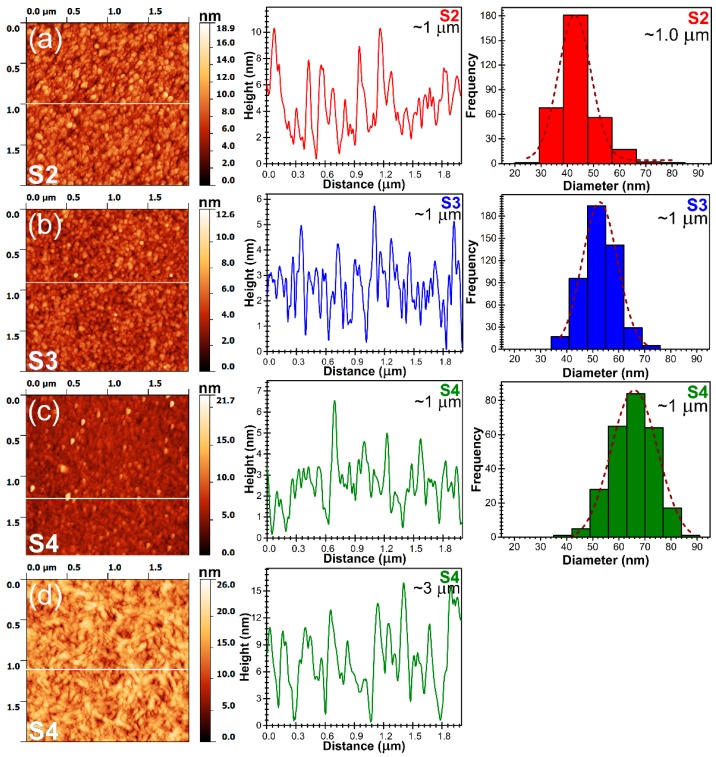
Characteristic atomic force microscopy (AFM) images, height profiles for the corresponding lines drawn in AFM images and grain size distribution histograms for AlN films deposited by reactive RF-MS starting from different base pressure levels: (**a**) S2 ≈ 1.0 μm, (**b**) S3 ≈ 1.0 μm, (**c**) S4 ≈ 1.0 μm and (**d**) S4 ≈ 3.0 μm.

The statistical grain size analysis was performed (in triplicate) for each type of sample using the SPIP 6.6.5 dedicated software on AFM (2 × 2 μm^2^) images. The mean grain size was extracted by Gaussian fitting of the frequency curves depicted by the midpoints of the histogram. Because a tip with good aspect ratio was used for AFM imaging, the lateral broadening effects was minimized. Of course, the broadening effects cannot be fully-suppressed, but the corresponding lateral distortion can be considered similar for all samples. Thus, the values and the general trend of the grain distributions presented in Figure 1 can be considered physically meaningful.

The average grain diameter has been found to increase with the film thickness, from ~65 nm (for a film thickness of ~1 μm) to ~90 nm (for a film thickness of ~3 μm), under the same deposition conditions (S4). At the same film thickness (i.e., ~1 μm), the average grain size increased with the decrease of the base pressure (contamination level) attained in the reactor chamber before deposition, from ~42 nm (S2), to ~52 nm (S3) and to ~65 nm (S4). 

The increase of surface R_RMS_ roughness with film thickness (Table 2), for the samples deposited in the same deposition conditions, can be pinpointed to the gradual improvement of films crystallinity and texture, which enables a progressive increase of the grain size and consequents in the coexistence at the film surface of grains at various development stages (Figure 1). On the other hand, it could be also noted a decrease of R_RMS_ (Table 2) with the decrease of the base pressure. Moreover, the films seem more compact (Figure 1) when the contamination level in the deposition chamber is lower (S2 < S3 < S4).

### 3.2. Structural Characterization

**Figure 2:**
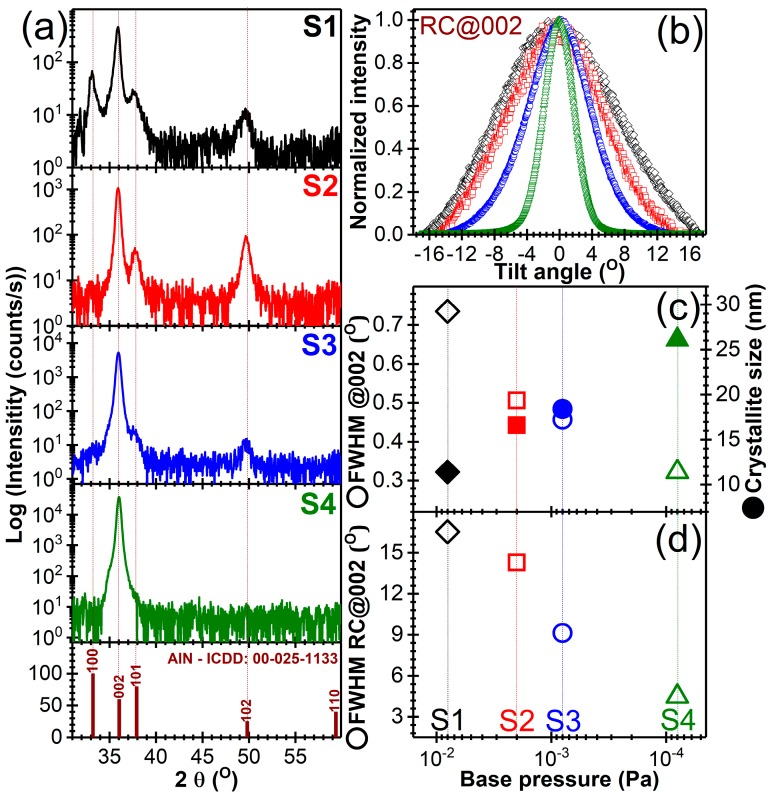
(**a**) X-Ray diffraction (XRD) diagrams (in logarithmic scale) of AlN films starting from different levels of base pressures (see Table 1). The ICDD reference file of hexagonal AlN is presented for comparison. (**b**) Comparative RCs recorded for the 002 reflections of AlN films. Evolution of the (**c**) FWHM of the 002 diffraction peaks (open symbols) and crystallite size along the *c*-axis (solid symbols) and (**d**) FWHM of the RCs of AlN (002) (open symbols).

**Figure 3:**
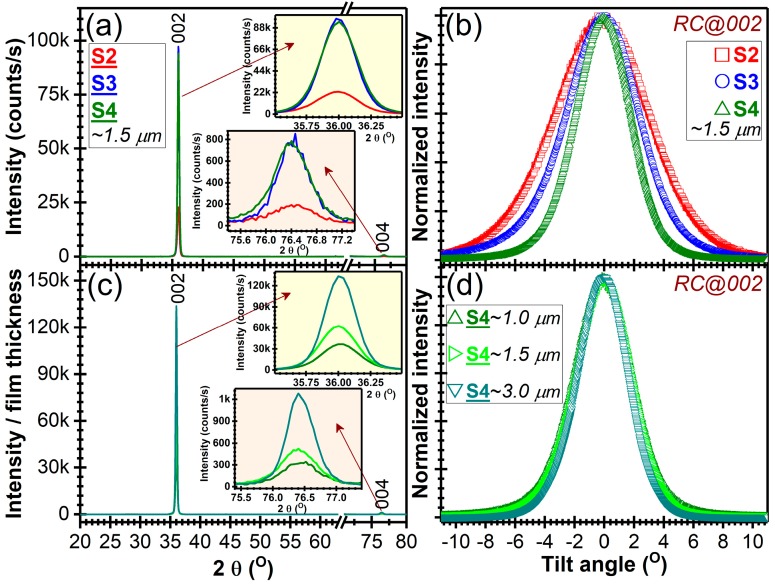
(**a**,**c**) XRD diagrams recorded in symmetric θ–θ geometry (**b**,**d**) RCs of the AlN 002 reflections. (**a**,**b**) Structure quality—base pressure dependence in the case of ~1.5 μm AlN films (**c**,**d**) Structure quality—thickness dependence in the case of S4-type films, synthetized starting from similar base pressure levels (see Table 1).

The X-ray diffraction analysis (symmetric θ–θ measurements and rocking curves) results are presented in Figure 2 and Figure 3. The ω-scan measurements (or rocking curves, RC) were performed in order to determine the degree of preferential orientation of the AlN films. The RC measurements provided information regarding the angular dispersion degree of the *c*-axis of hexagonal AlN crystallites with respect to the normal to the substrate. A reduced FWHM of the RCs indicates a high degree of preferential orientation of the crystallites.

At the same thickness, the films crystallinity improved with decreasing base pressure attained in the reactor chamber prior to introducing the high purity (6N) working/reaction gases (Figure 2). A low base pressure is generally associated with the low contamination levels (e.g., hydrocarbons or oxygen residual molecules, water vapors or other volatiles compounds). Such contaminants might interfere with Al–N reaction processes taking place at the substrate, or even might get incorporated in the growing film. Ultimately, such undesirable events can lead to generation of defects, which will be further transmitted upwards leading to a defective film structure with low degree of ordering (crystallinity).

By reducing the quality of the base pressure conditions [from ~6 × 10^−5^ Pa (S4) to ~6 × 10^−3^ Pa (S2)], the film *c*-axis texturing is strongly reduced (Figure 2b), besides the 002 peak being highlighted also the presence of other reflections (i.e., 100, 101, 102) of an AlN phase crystallized in the hexagonal system (ICDD: 00-025-1133) (Figure 2a). 

Furthermore, the gradual decrease of the FWHM values of the 002 diffraction peaks (Figure 2c and Figure 3, Table 3), indicative of their crystalline quality, occurred with (i) the reduction of the base pressure and (ii) the increase of the films thickness. A similar trend has been depicted by the FWHM of the RCs (Figure 2c and Figure 3, Table 3), denoting the progressive texturing of the AlN films with both their thickness and the initial base pressure quality. The average crystalline coherence length (“crystallite size”) along the *c*-axis was estimated from the FWHM of 002 diffraction line applying the Scherrer equation. The line width was corrected for instrumental line broadening using a corundum NIST standard reference material 1976. The 104 corundum line was used (positioned at 2θ ≈ 35.12°) having a FWHM of 0.198° with the X-ray optics employed in the current study. The crystallite size of the AlN films increased progressively with the diminution of the base pressure, reaching a maximum value of ~26 nm (Figure 2c).

The 1.5 µm-thick AlN films showed a more pronounced decrease of FWHM of RCs with base pressure, with respect to the 1 µm thick ones (Figure 3a,b, Table 3). This suggests that the magnetron gun might act as ion getter pump during a prolonged deposition, with the ionized residual molecules being attracted and then gradually consumed/adsorbed/reacted at the surface of the cathode, thereby leading in time to a reduction of the contamination level. This might be considered as a second factor that determines the progressive alignment of *c*-axis crystallites with the film thickness increase (longer deposition time), besides the Wolmer-Webber growth mechanism, specific to the thermodynamics of the employed sputtering process, which fosters the gradual alignment of the crystallites along the normal to the substrate (in the direction of the sputtered atoms flux). Under the low temperature sputtering conditions, the ad-atoms possess reduced transverse surface mobility, which makes them to frozen at their arrival sites, leading to the rapid growth in the [002] direction, and hinders the development of crystallites with other orientations. For the ultimate base pressure, attained with the employed deposition system, a progressive monotonous improvement of both crystallinity and texturing degree was noticed (Figure 3c,d, Table 3) with film thickness increase from ~1 μm to ~3 μm (i.e., the FWHM of the RCs reached a value of ~3.9°).

Typically, superior *c*-axis texturing results (FWHM of RC < 0.5–2°) are obtained when employing high powers on cathode target [44,47,72,73], which implies intense bombarding processes and consequently a higher deposition temperature (>200 °C), detrimental for heat-sensitive substrates. When the deposition temperature is lower, the ad-atoms arriving at the substrate surface have reduced traverse mobility which impedes a high ordering and texturing of the growing AlN film. Usually, in such situations poorly or non-textured films are obtained [74,75]. The FWHM value of the RC obtained in the case of S4 samples (i.e., ~4.5°) is consistent with the ones obtained at a similar deposition temperature by Akiyama et al. [73] or Yarar et al. [76]. A FHWM of RC of ~2.3°, in the case of AlN film deposited on Si substrates, was obtained at relatively low temperature (~150 °C) by Naik et al. [77], but as the authors stated that the decisive condition for attaining such high *c*-axis texturing was the low base pressure (i.e., 6.6 × 10^−8^ Pa).

A high *c*-axis texturing of AlN films is viewed a prerequisite for piezoelectric properties. Films with FWHM of RC (002) of ~8° with piezoelectric response are often reported [78,79,80]. Furthermore, Akiyama et al. [79] obtained such AlN films which exhibited piezoelectric response over a wide temperature range (i.e., −196 to 300 °C), furthermore being able to measure pressure from pulse waves of hundreds of pascals to 40 MPa. Sanz-Hervás et al. [78] and Jackson et al. [80] have indicated that decisive for the functional properties of such AlN films is in fact the absence of traces other crystalline plane reflections besides the ones parallel to the surface.

These results were confirmed also by our previous experiments with AlN films of similar texturing degree, as AlN films were successfully integrated in SAW resonators which delivered excellent frequency response [81] or elicited promising pyroelectric properties [15].

Moreover, the AlN films with lower crystallinity and poorer texture could serve as active layer in other applications of high interest: gate-dielectrics in thin films transistors [82] or metal-insulator-metal capacitors on both rigid and flexible substrates. 

**Figure 4:**
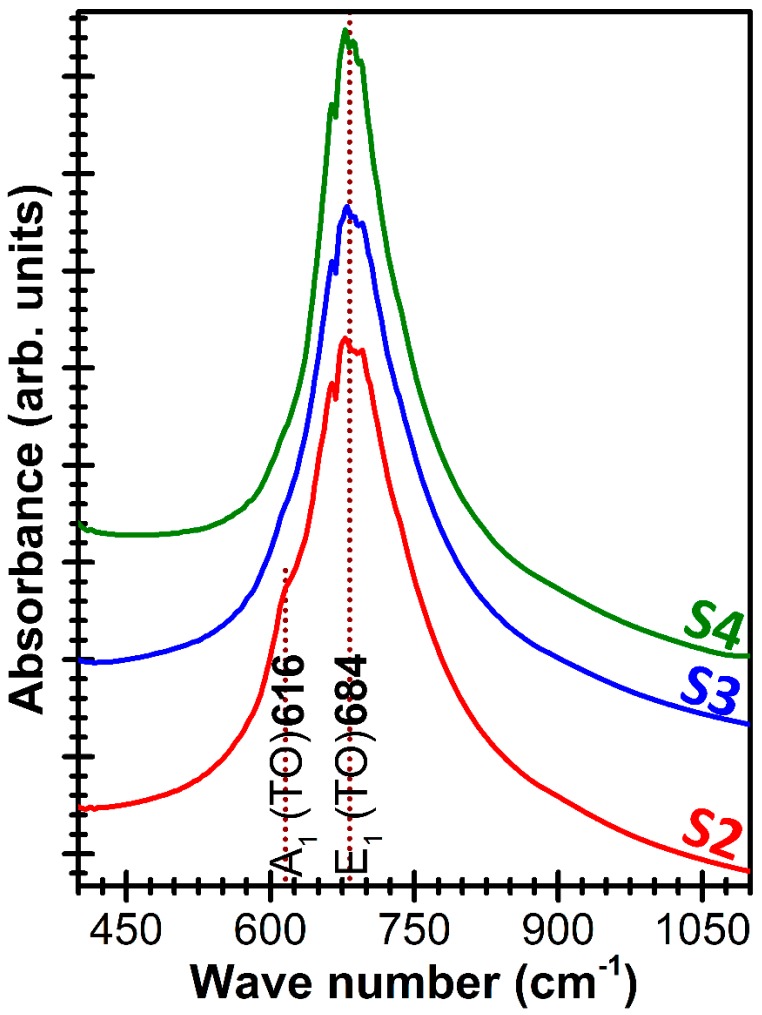
Comparative Fourier Transform Infrared (FTIR) spectra of ~1 μm thick AlN films deposited starting from different base pressure levels.

The FTIR spectra displayed the characteristic vibration modes of an AlN wurtzitic structure (Figure 4) [83]. The band centered at ~684 cm^−1^ is attributed to the IR active vibration modes of the transversal optical polar phonons (E_1_ (TO)). In the case of the films with a reduced *c*-axis texturing (e.g., S2) a supplementary vibration band could be also observed at ~616 cm^−1^, as a low intensity shoulder. This band is associated in literature with transversal optical phonon modes (A_1_ (TO)) [84,85,86]. In the case of structural vibrations with A_1_ (TO) and E_1_ (TO) symmetry, the atoms are moving parallel and perpendicular to the *c*-axis, respectively. The emergence of this new vibration (A_1_ (TO)) mode could be due to the increased tilting angle of the crystallites from the ideal *c*-axis orientation [83].

**Figure 5:**
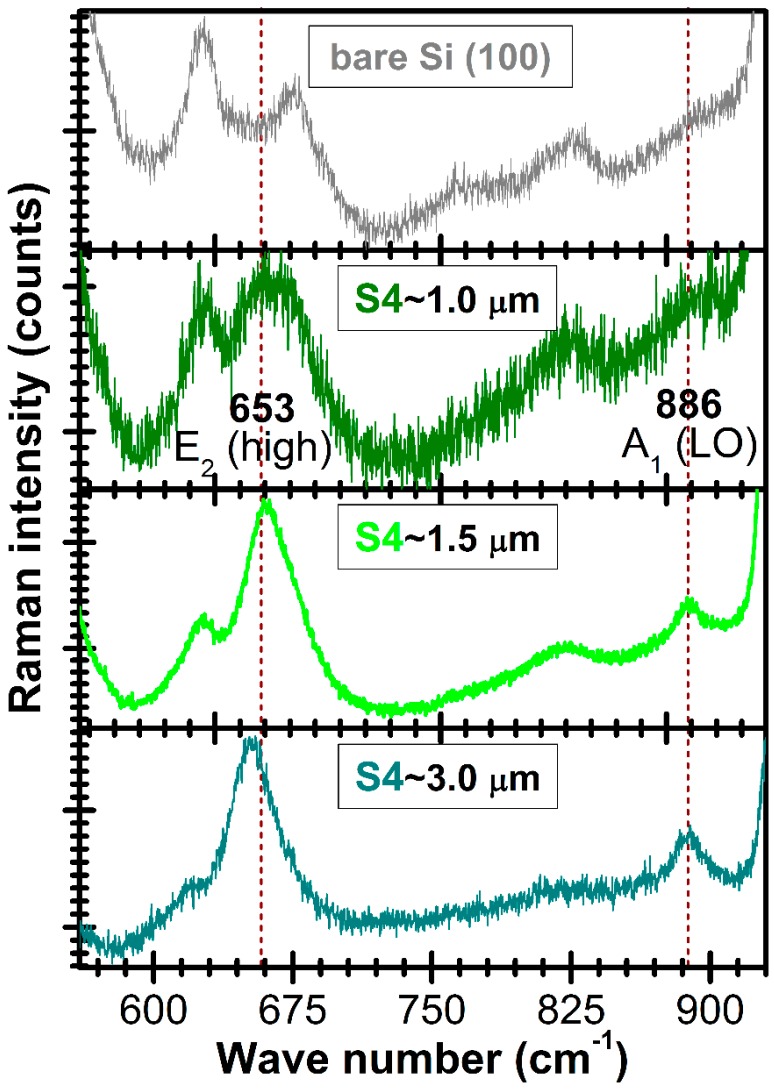
Comparative micro-Raman spectra of the S4—AlN/Si samples (with different film thicknesses) and of the bare Si substrate.

The comparative micro-Raman spectra of the AlN/Si (100) films having different thicknesses and of the bare Si (100) substrate are shown in Figure 5. In the case of this type of measurements, the film thickness is very important because the Raman signal is conditioned by a sufficient scattering volume. In the case of AlN film with a thickness of ~1 μm, the dominant vibration modes are those of the Si substrate. When increasing the film thickness, the intensities of Raman phonon modes E_2_ (high) ~653 cm^−1^ and A_1_ (LO) ~886 cm^−1^ (see Table 4) are progressively increasing as well. The advent of the two Raman maxima is typical for AlN films with strong *c*-axis texturing [84,87,88,89] and is in good agreement with the XRD investigations (Figure 2 and Figure 3).

### 3.3. Compositional Investigation

**Figure 6:**
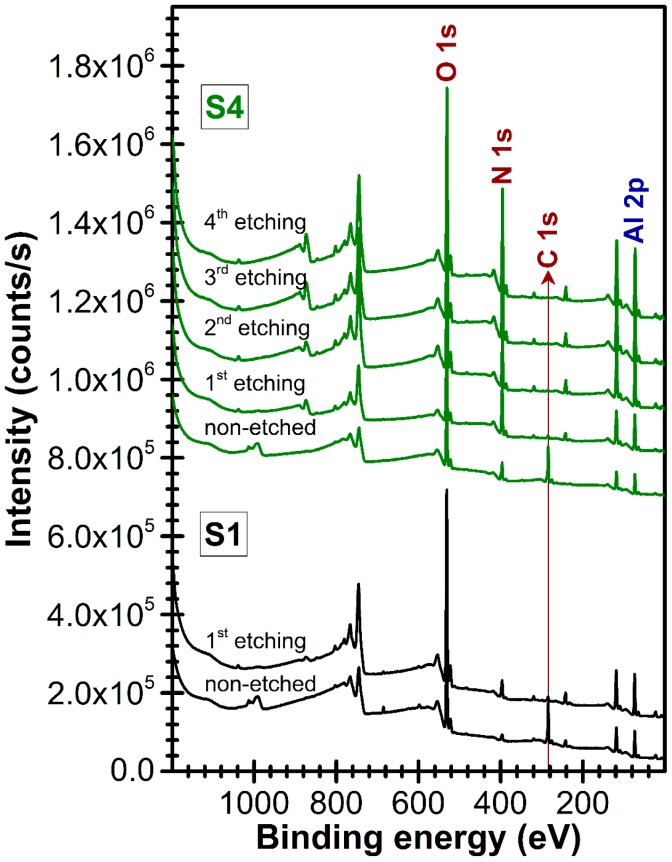
Comparative XPS spectra of S1 and S4 films, before and after the surface argon ion etching stages.

**Figure 7:**
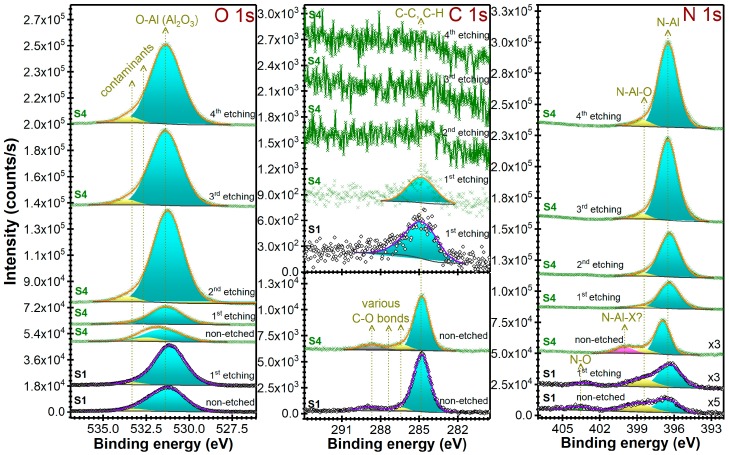
High-resolution XPS spectra of O 1s, C 1s and N 1s levels of S1 and S4 films, before and after the surface argon ion etching stages. Symbols—experimental data, Solid lines—peaks fit sum and individual identified components.

For the identification of contaminants nature and estimation of contamination levels, XPS analyses have been carried out (Figure 6 and Figure 7) on (~1 μm thick) AlN films.

The binding energy of N 1s photoelectron level in the case of Al–N bonds ranges from 396 eV to 398.5 eV. The energy of Al–N–O bonds is 2–3 eV larger, with further oxidation leading to higher binding energies. The wide spread of energy values is determined by several factors: spectrometer type, contamination configuration at the sample surface, bonding configuration, surface morphology and chemical environment where the AlN is found. Furthermore, it should be pointed out that after ion etching, one no longer has an energy reference since C is no longer present at the surface. Our peak position fits very well with other similar experiments reported in the literature [61,90].

In the case of the non-etched sample, the fairly large hump situated at ~990 eV appertains to the K-inner level-valence-valence electron emission (KVV) Auger line of C (Figure 6). After argon ion etching the Auger line of C disappears, but a L-inner level-M-inner level-M-inner level electron emission (LMM) Auger line of Ar becomes visible at ~1039 eV. The Ar is implanted during the etching process. This is supported also by the presence of other specific lines: Ar 2p situated at 242 eV and Ar 2s situated at 320 eV.

It was found that carbon based contaminants are present only at the surface of the film. By applying a mild argon ion etching step (that removes ~1–2 nm from the film surface), a strong decrease of films carbon content, accompanied by the increase of nitrogen one, was noticed.

AlN films contain also oxygen, which is present in the entire film’s volume (Figure 6). The structural difference between the S1 sample (with weakest *c*-axis texturing) and the S4 one (with highest *c*-axis texturing) is clearly influenced by the oxygen contamination level. After the first argon ion etching step, the N 1s core electron level line of S1 indicated the presence of three components having different weights: Al–N bonds = 66.4%, Al–N–O bonds = 27.1% and N–O bonds = 6.5% (Figure 7); whist in the case of S4 two components have been evidenced: Al–N (94.4%) and Al–N–O (5.6%) bonds (Figure 7). The further depth profile XPS compositional studies (Figure 7) performed in the case of S4 sample did not revealed substantial concentration modifications in the film volume, the Al–N bonds content varying in the narrow range 94.4–95.6%, with Al–N–O bonds as minuend. The presence of oxygen and absence of carbon (at the sensitivity limit of the XPS machine) suggests that water vapors are the main source of reactor chamber contamination, governing the level of the base pressure.

### 3.4. Mechanical Testing

#### 3.4.1. Pull-Out Testing

For appraisal of tensile strength of the epoxy adhesive, the stainless steel testing elements were glued directly onto the bare Si substrates. The failure was of cohesive type, as the fracture occurred at values of 75.1 ± 4.3 MPa in the adhesive volume (and not at the interface with the substrate), this being further considered as its mechanical resistance limit.

The adherence of the AlN film to the substrate is an indispensable factor, as, in many respects, determines the lifetime/sustainability of the optoelectronic devices in which will be integrated. The delamination of the active/functional film is the prominent critical failure mode of such devices, due to inadequate film/substrate bonding strength.

**Figure 8:**
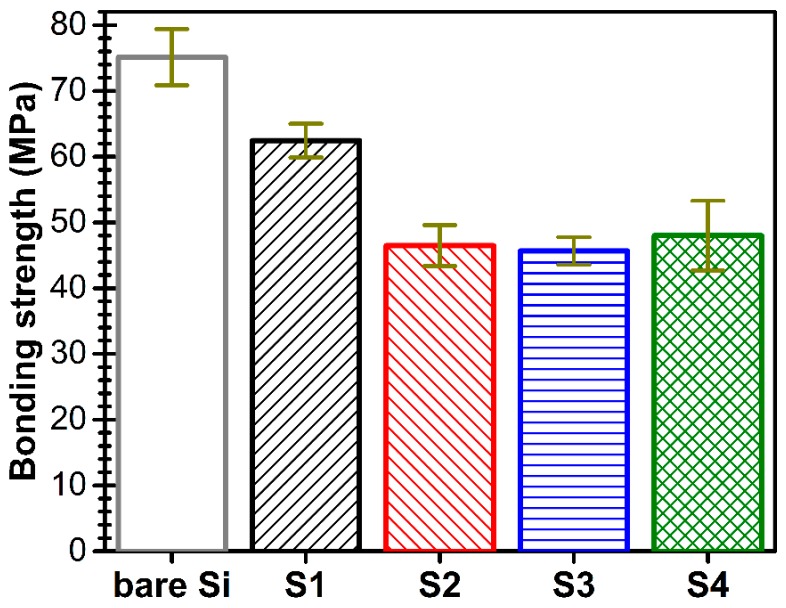
Pull-out bonding strength values for AlN films with various degrees of *c*-axis texturing.

The pull-out tests were comparatively performed for (~1 μm thick) AlN films with various degrees of *c*-axis preferential orientation (i.e., S1, S2, S3 and S4). Remarkable bonding strength values have been obtained, in the range of 45–62 MPa (Figure 8), the films detachment/failure being of adhesive type and thus taking place each time at the AlN film/Si substrate interface.

The unpaired Student *t*’test statistical analysis (assuming unequal variances) indicated significant statistical differences (*p* < 0.05) only between S1 films and the other type of the samples. No statistical significant differences were evidenced between S2, S3 and S4 coatings.

#### 3.4.2. Nano-Indentation, Nano-Scratch and Nano-Wear Tests

**Figure 9:**
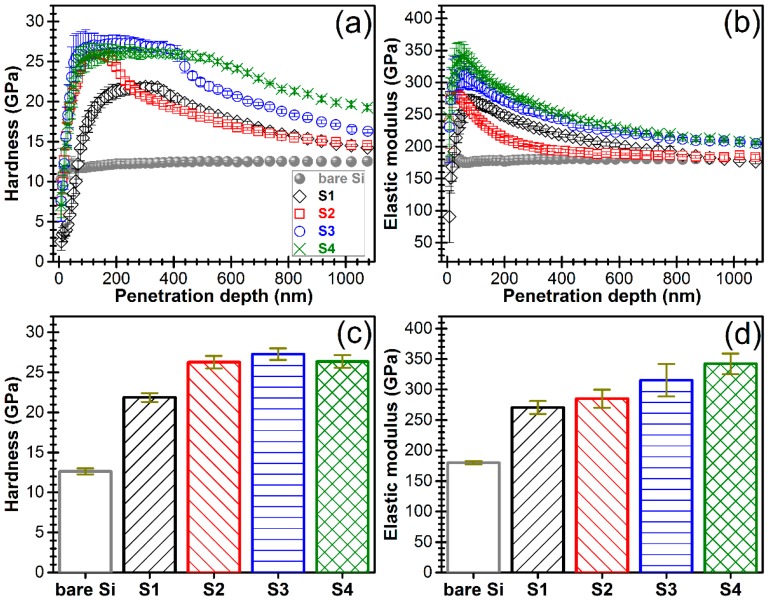
(**a**) Hardness and (**b**) elastic modulus vs. the penetration depth in the case of AlN films with various degrees of *c*-axis texturing. Graphs depicting the evolution of the (**c**) hardness and (**d**) elastic modulus average values of the AlN films.

The nano-mechanical tests (Figure 9) revealed an overall enhancement of the mechanical properties of the (~1 μm) AlN films with their improved purity (Figure 7) and structural quality (Figure 2). In the case of the AlN film with the highest crystalline quality and *c*-axis texturing (i.e., S4) the elastic modulus values (342.3 ± 16.9 GPa) are comparable with the highest values reported for the compact AlN pellets (302–348 GPa) [91]. Furthermore, for this type of film, the hardness values (26.4 ± 0.8 GPa) are higher than those reported in literature for bulk AlN ceramics (9.9–12 GPa) [91]. The AlN film with the lowest crystallinity status and *c*-axis texturing (i.e., S1) elicited elastic modulus (270.6 ± 10.6 GPa) and hardness (21.9 ± 0.54 GPa) values similar to the ones obtained by Yang et al. [50] (E ≈ 242 GPa and H ≈ 19 GPa) for an AlN sputtered film with similar structural properties. 

**Figure 10:**
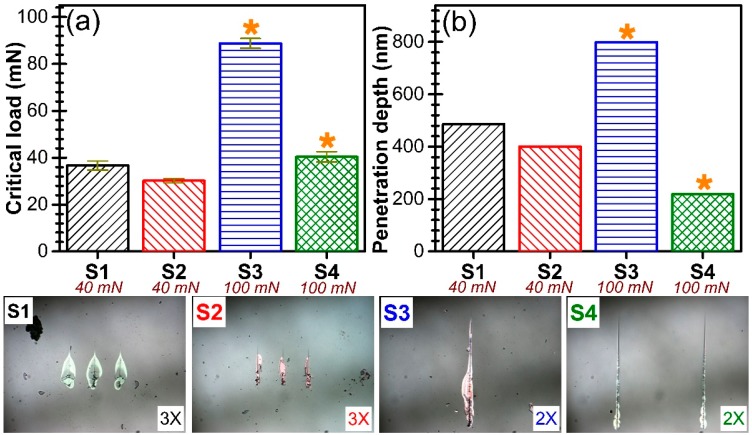
Graphical representation of the (**a**) critical load values at which destructive events occurred and (**b**) the corresponding penetration depth values. Microscopic images of the scratches are enclosed.

The nano-scratch tests were done first using incremental loads up to a maximum value of 40 mN. In this case, destructive events were recorded only for the S1 and S2-type samples, at critical loads of 36.6 ± 1.9 mN and 30.2 ± 0.9 mN (Figure 10a), respectively; the registered penetration depths were of ~490 nm and ~400 nm, respectively (Figure 10b). For the S3 and S4-type films, having a superior crystalline quality and higher *c*-axis texturing, no destructive events were recorded for load values up to 40 mN. Further, for these type of films only, were applied incremental loads to a maximum value of 100 mN. Still, no failure has been noticed. It should be noted that the critical load values shown in Figure 10a are not the true ones, the delamination of the S3 and S4 samples occurring only at the extraction of the indentation tip from the films. The penetration depths at the 100 mN load in the case of the S3 and S4 films was of ~790 nm and ~218 nm, respectively (Figure 10b). These values confirm the excellent mechanical quality of this type of AlN films.

**Figure 11:**
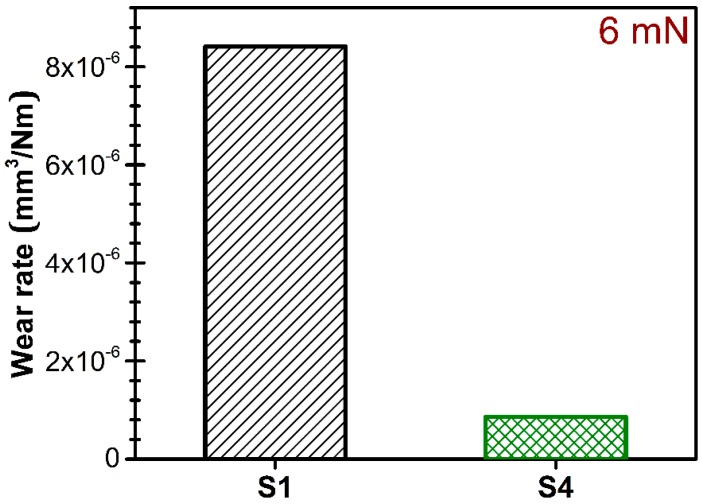
Graphical representation of the wear rate values of the S1 and S4 films obtained by reciprocating sliding tests carried out at a force of 6 mN for 400 cycles.

Moreover, the wear resistance of highly textured (S4) AlN films can be emphasized, the recorded wear rate being of ~8.6 × 10^−7^ mm^3^/Nm at a 6 mN load (Figure 11). The survey of the scarce literature available on this topic revealed similar nano-scratch test responses (Jian et al. [92] reporting no evidence of phase transformation or cracking up to the maximum load of 80 mN) in the case of AlN films, and superior wear performance with respect to AlTiN films (Nohava et al. [93] obtaining a higher wear rate of ~20 × 10^−7^ mm^3^/Nm).

### 3.5. Corrosion Tests

**Figure 12:**
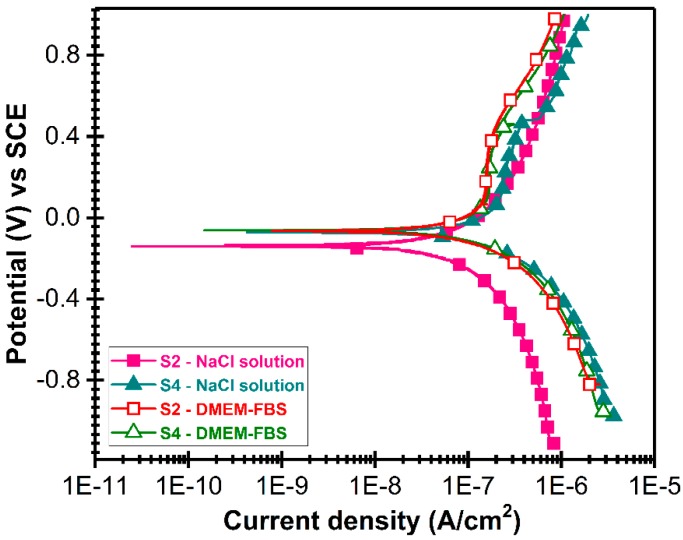
Evolution of potentiodynamic curves recorded in the case of S2 and S4-type AlN films, tested in NaCl and DMEM-FBS solutions, respectively.

The S2 and S4-type samples, with highly dissimilar structural features, were chosen for corrosion testing. The potentiodynamic curves recorded for these two types of AlN samples are presented comparatively in Figure 12. The main extracted electrochemical parameters are showed in Table 5.

As it is commonly admitted, electropositive E_corr_, low i_corr_ and high R_p_ values indicate a high resistance to the corrosive attack of corrosive solutions [94,95]. If we take into account these criteria, it can be seen that S4-type sample is more resistant in NaCl solution than S2-type sample. In the DMEM-FBS solution, S4-type sample exhibited lower corrosion current density and corrosion rate, while S2-type sample showed higher values of polarization resistance and a more positive value of the corrosion potential. This effect may be related to the fact that S4-type sample has a compact morphology (Figure 1). Nevertheless, this behavior is more difficult to be explained, since it is generally admitted that one of the most important factors influencing the degradation of implanted medical devices is the degradation rate in contact with body fluids. Therefore, it is reasonable to suppose that for the biomedical applications is more important to have a low corrosion rate than high polarization resistance. Based on this, we can consider that the S4-type sample has a superior corrosion resistance in DMEM-FBS solution compared with S2-type sample. According to the potentiodynamic curves, S4-type samples presented some fluctuations during the entire immersion period, hinting to possible dissolution processes of oxidized zones due to the chloride attack and penetration of electrolyte into the pores of coatings.

The corrosion rate is significantly influenced by both the AlN structural quality and type of testing medium (Table 5). The corrosion rates of S2-type samples (having lower crystallinity and decreased texturing degree) were (i) ~9.5 and (ii) ~48 times higher with respect with the values elicited by the S4-type samples when testing in (i) the purely inorganic NaCl 0.9% saline solution and (ii) the medium with high degree of biomimicry (DMEM-FBS), respectively. One can observe, that the corrosion rates drop ~2 times in the case of S2-type samples and ~10 times in the case of S4-type sample, when using a medium which better mimics the true intercellular fluid composition instead of the classical NaCl saline solution. Thus, the results are suggesting that the crystalline quality of the AlN films is a parameter of paramount importance in view of developing highly sustainable electronic device designs capable to work in contact with aqueous biological media for prolonged periods of time. The lower corrosion rates recorded in the case of DMEM-FBS tested samples could be associated with the formation of a protective layer by adsorption processes of proteins and other organic moieties at the AlN sample surface, as observed in the case of bioglass coatings tested in vitro in the same type of medium [70]. Such a protein capping layer could hinder the corrosion process of AlN films. The corrosion of AlN in aqueous media relies on hydrolysis processes. Bowder et al. [96] was the first to report the kinetics of AlN (powders) hydrolysis, identifying the following scheme of reactions thermodynamically favorable at RT:

AlN + 2H_2_O → AlOOH_amorph_ + NH_3_ (a)

NH_3_ + H_2_O → NH_4_^+^OH^−^ (b)

AlOOH + H_2_O → Al(OH)_3_ (c)

Furthermore, Krnel et al. [97] and Reetz et al. [98] indicated that these processes are accelerated with the increase of pH and temperature, whereas at very low pH (pH ≈ 1) the hydrolysis of AlN is virtually prevented. The samples of AlN that undergo biocompatibility testing (being maintained in an atmosphere with a 5000 Pa partial pressure of CO_2_, at a constant pH of around 7.4) elicit a slower degradation rate in contrast to samples tested in inorganic solutions used for various degradation tests (that tend to become more basic in normal atmosphere and therefore to increase corrosion phenomena).

The higher corrosion rate recorded in saline solution for the S2-type samples deposited by reactive RF-MS (12.93 µm/year) is close to the lowest ones reported by Jatisukamto et al. (12.93 µm/year) in the case of AlN films deposited by reactive direct-current MS [99]. The ~0.13 µm/year (~0.3 nm/day) corrosion rate recorded for S4-type sample, suggests that for AlN-based sensor devices, designed to be operated in situ in the human body, precautions should be taken into account; for instance, by application of encapsulation procedures. Even though the AlN corrosion products do not seem to have a cytotoxic effect on living cells (as shown further), the functionality of the electronic devices will be seriously affected by the continuous surface modification of the AlN layer. To our opinion this requires emphasizing, as previous reports have advanced AlN as a feasible solution for developing biosensors, but no reports so far, to the best of our knowledge, have stressed the possible deleterious effect of the corrosion of AlN in aqueous biological media.

### 3.6. Biocompatibility Assays

#### 3.6.1. Cell Cultures

The cytocompatibility of AlN films was assessed using an Hs27 fibroblasts cell line. The phenotype choice is justified by the fact that the tissue repair is primarily realized by fibroblast cells and only secondary by the specialized cells typical to each type of tissue.

#### 3.6.2. Cell Morphology

Since the AlN/Si samples were opaque, the morphology of cells grown on the bare Si substrate and (~1 μm thick) AlN films with dissimilar *c*-axis texturing degree (i.e., S2 and S4) has been investigated by epi-fluorescence microscopy. The cells were stained with phalloidin-AlexaFluor546 fluorochrome (red labelling) to reveal the actin microfilaments of the cells cytoskeleton. Any cellular morphological changes will be reflected in the actin cytoskeleton alterations. The cells nuclei were counterstained with DAPI fluorochrome (blue labelling) to unveil their shape and any eventual apoptotic chromatin condensations.

**Figure 13:**
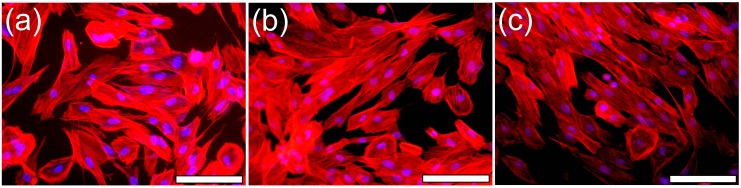
Epi-fluorescence microscopy images revealing the morphology of Hs27 fibroblast cells grown on: (**a**) bare Si substrate and (**b**) S2 and (**c**) S4 type films. The actin cytoskeleton was stained with phalloidin-AlexaFluor596 (red), whilst cell nuclei were counterstained with DAPI (blue). Objective: 20X. Magnification bar: 50 μm.

In the case of both the bare Si substrate and AlN films, the Hs27 fibroblast cells adhered rapidly to the surface and presented a normal morphology (Figure 13). The observed shape and dimensions are characteristic to the Hs27 cell line. At 24 h after seeding, the cells were well-spread, eliciting a normal fusiform polygonal-like aspect. In all the cases were revealed ovoid cells nuclei without pathological chromatin condensation. Thus, the cells morphology advocate for the biocompatibility of AlN films synthesized by reactive RF-MS.

#### 3.6.3. Cells Proliferation

**Figure 14:**
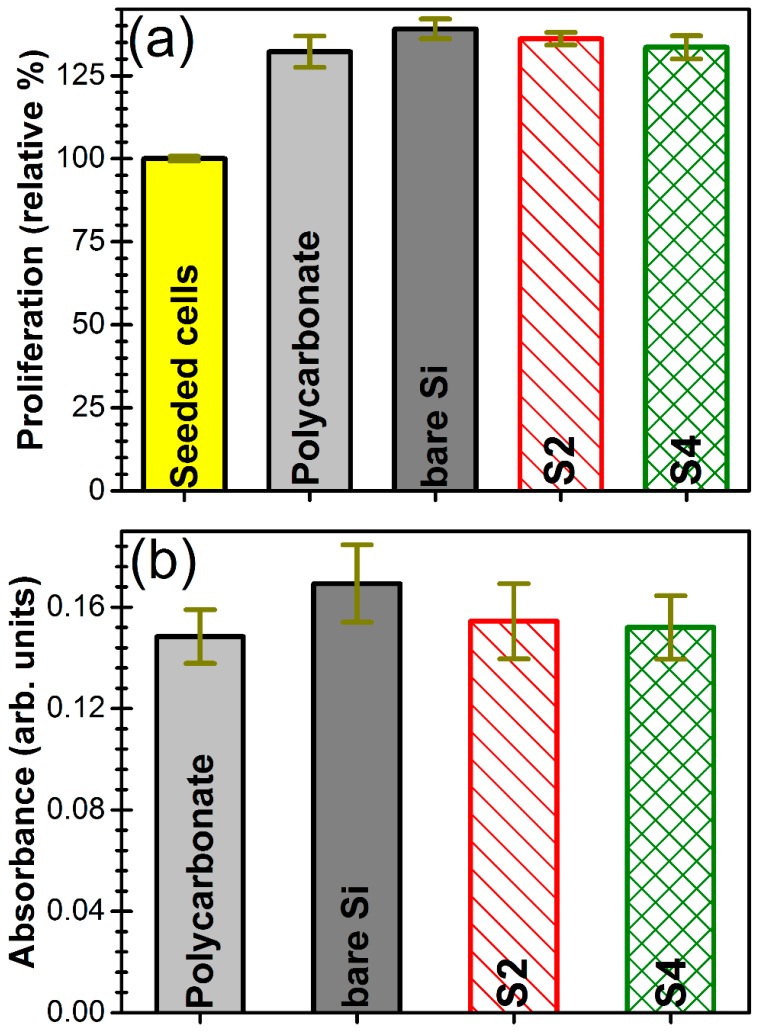
Histograms displaying (**a**) the Hs27 cell proliferation results as obtained by the MTS assay (The MTS values are normalized as the percent to the absorption of the seeding cell number) and (**b**) Cytotoxicity of samples as revealed by LDH assay after 24 h of culturing (The LDH values are presented in arbitrary absorption units, showing the amount of dead cells).

The cells proliferation has been tested by the MTS assay. In Figure 14a are presented the results obtained 24 h after cells seeding on different sample surfaces: polycarbonate biological control, bare Si substrate, S2 and S4 type AlN films. The recorded proliferation was of ~35%, with no significant differences (*p* > 0.05) between the studied cases. Thus, the MTS experiments indicated the excellent proliferation of the fibroblast cells, which supports the good cytocompatibility of AlN films, regardless of their *c*-axis texturing degree.

#### 3.6.4. Cells Viability/Cells Death

The cytotoxicity of the AlN films has been quantified by assessing the activity of the LDH enzyme in the cell culture media. LDH is an intracellular enzyme found in all cells. When a cell dies, LDH is released into the cell culture medium. Therefore, the LDH activity in the cell culture media is proportional to the number of dead cells. The obtained data were compared to a previously determined scale concerning the typical quantity of LDH enzyme in the Hs27 cell line. Further, the number of dead cells was estimated by normalization to the total number of cells recorded (24 h after seeding) for each studied situation.

The results presented din Figure 14b indicated the excellent biocompatibility of AlN films. In all studied cases, the number of dead cells was similar (with no significant differences), with the overall cellular mortality situated under 2%.

Even if according to the suggested corrosion mechanism, ammonia would form in the cell growing media upon AlN corrosion, at the rates indicated by the dissolution tests, one would obtain around 10 µg/dL in the case of S4 sample and 500 µg/dL for the S2 one. Part of the ammonia resulted in the corrosion would be transferred in the atmosphere and some would interact with the HCO_3_^−^ and also with amino acids found in the solution, creating complexes as amides, which diminishes their toxicity. In newborns the ammonia concentration is around 90–150 µg/dL [100] and medical aggressive approach (blood dialysis) is taken when ammonia concentrations are greater than 1000 µg/dL [101]. Therefore, the excellent biocompatibility of AlN films could be a result of the DMEM-FBS mechanisms of mitigating the free ammonia effects.

## 4. Conclusions

The sputtering variables have been chosen based on experience and relevant literature to accommodate the synthesis of *c*-axis textured AlN films. By employing a low target power density (~0.008 W/mm^2^) and relative short target-to-substrate separation distance (30 mm), the fabrication of good quality AlN films at low substrate temperature (~50 °C) and high deposition rate (~14 nm/min) becomes feasible, thus making the process economically attractive for flexible electronics. 

The base pressure attained in the reactor chamber prior to working gas admittance was found to play a role of paramount importance for obtaining *c*-axis textured AlN films. The XRD, FTIR and micro-Raman spectroscopy measurement evidenced the hexagonal structure of AlN films, eliciting various degrees of *c*-axis texturing, as a function of the base pressure level attained in the reactor chamber prior to the introduction of the working/reactive gases. 

XPS investigations were performed on AlN films with different structural qualities in order to identify the influential factors which hinder their crystallization and *c*-axis texturing. It was revealed that the main difference between a highly crystallized AlN film with pronounced *c*-axis texture and an AlN film with inferior structural quality lies in their residual oxygen content.

The best results (FWHM of 002 rocking curves of ~4.5° at a film thickness of 1 μm) were obtained starting from the ultimate base pressure (~6 × 10^−5^ Pa) attainable with the employed deposition system. The texturing degree is fairly similar to the one reported by Akiyama et al. [73] or Yarar et al. [76], at a comparable deposition temperature.

Our AlN films presented remarkable multi-parametric qualities: compact morphology, low roughness (~1.5 nm), low oxygen content and absence of carbon or carbonaceous compounds in its volume, very good adherence to the substrate (~47.6 ± 2.6 MPa), elastic modulus close to the one of bulk ceramic (342.3 ± 16.9 GPa), superior hardness compared to AlN bulk ceramics (26.4 ± 0.8 GPa) and excellent scratch and wear resistance (wear rate at 6 mN: ~8.6 × 10^−7^ mm^3^/Nm). The mechanical performance is in many respects superior to results generally reported in literature.

The corrosion assays, relevant in view of developing in vivo sustainable devices, were performed in both saline solution and DMEM-FBS biomimetic medium. The results emphasized the superior performance of the well-crystallized and highly *c*-axis textured AlN films. A corrosion rate of ~0.13 µm/year was deduced for such AlN structures.

Furthermore, the study brings new insights on a subject rarely approached in literature: the cytotoxicity of AlN films. The in vitro tests in fibroblast cell (Hs27) cultures endorsed the cytocompatibility of AlN thin films, regardless of their structural quality, allowing good adherence, healthy morphology and great proliferation of the cells (with respect to both bare Si substrate and polycarbonate biological control). The cellular mortality index, determined by quantifying the activity of the LDH enzyme, is comparable to control, having values below 2% from the total cell number.

The inter-disciplinary results gathered in the present study suggest that good quality AlN films could be achieved even at a low substrate temperature. Nevertheless, although the AlN corrosion products do not seem to influence the cells adherence, viability and proliferation, the functionality of AlN-based electronic devices to be used in prolonged contact with body fluids in vivo needs to be questioned. Whereby in such conditions the continuous surface modification of the AlN layer will definitely affect the overall performance, remedy solutions need to be identified and integrated (e.g., device encapsulation with biocompatible non-degradable compounds), in order to safely and fully exploit the remarkable potential of AlN films for biosensors and implantable sensors.

## Figures and Tables

**Table 1:** Aluminum Nitride (AlN) thin films deposition conditions.

Sample Code	Base Pressure (Pa)	Total Working Pressure (Pa)	Nitrogen Partial Pressure (Pa)	Target-to-Substrate Distance (mm)	Film Thickness (μm)
S1	~6 × 10^−3^	0.2	0.06	30	~1
S2	~2 × 10^−3^	0.2	0.06	30	~1; 1.5
S3	~8 × 10^−4^	0.2	0.06	30	~1; 1.5
S4	~6 × 10^−5^	0.2	0.06	30	~1; 1.5; 3

**Table 2:** Root mean square roughness (R_RMS_) values inferred from the AFM measurements performed on 2 μm × 2 μm microscopic fields for AlN films deposited under different working conditions.

Sample Code	Film Thickness (μm)	R_RMS_ (nm)
S2	~1.0	~2.2
S3	~1.0	~1.3
S4	~1.0	~1.4
S4	~3.0	~3.4

**Table 3:** FWHM values of the 002 diffraction peaks and their corresponding RCs.

Sample Code/Thickness	FWHM 002 (°)	FWHM RC@002 (°)
S1/1.0 μm	0.73	16.5
S2/1.0 μm	0.51	14.3
S3/1.0 μm	0.46	9.1
S4/1.0 μm	0.32	4.5
S2/1.5 μm	0.35	7.5
S3/1.5 μm	0.31	5.8
S4/1.5 μm	0.29	4.3
S4/3.0 μm	0.26	3.9

**Table 4:** The characteristic IR active/inactive phonon modes of aluminum nitride [84,88,89].

Wave Number (cm^−1^)	Phonon Mode	IR Active/Inactive
248	E_2_ (low)	inactive
610	A_1_ (TO)	active
655	E_2_ (high)	inactive
667	E_1_ (TO)	active
888	A_1_ (LO)	active
910	E_1_ (LO)	active

**Table 5:** Polarization resistance (R_p_), corrosion potential (E_corr_), corrosion current density (i_corr_) and corrosion rates (C_rr_) recorded for S2 and S4-type AlN films in NaCl saline solution and DMEM-FBS biomimetic medium.

Sample Type	R_P_ (kΩ)	E_corr_ (mV)	i_corr_ (μA)	C_rr_ (μm/year)
NaCl testing solution				
S2	160.06	−118.70	0.9428	12.93
S4	304.18	−38.60	0.0993	1.36
DMEM-FBS testing solution				
S2	474.08	−34.90	0.4631	6.35
S4	350.06	−110.10	0.0094	0.13
